# Correlation between early features and prognosis of symptomatic COVID-19 discharged patients in Hunan, China

**DOI:** 10.1038/s41598-021-83654-7

**Published:** 2021-02-22

**Authors:** Yeyu Cai, Jiayi Liu, Haitao Yang, Taili Chen, Qizhi Yu, Juan Chen, Deng Huang, Zhu Chen, Quan-Liang Shang, Cong Ma, Xiangyu Chen, Enhua Xiao

**Affiliations:** 1 Department of Radiology, The Second Xiangya Hospital, Central South University, Changsha, 410011 Hunan Province China; 2 Department of Oncology, Xiangya Hospital, Central South University, Changsha, Hunan Province China; 3 Department of Radiology, The First Hospital of Changsha, Changsha, Hunan Province China; 4 Department of Radiology, The Central Hospital of Xiangtan, Xiangtan, Hunan Province China; 5 Department of Respiratory Medicine, Yangxin County People’s Hospital, Huangshi, Hubei Province China; 6 Molecular Imaging Research Center, Central South University, Changsha, 410011 Hunan Province China

**Keywords:** Diseases, Medical research

## Abstract

To determine the correlation between the clinical, laboratory, and radiological findings and the hospitalization days in Coronavirus Infectious Disease-19 (COVID-19) discharged patients. We retrospectively identified 172 discharged patients with COVID-19 pneumonia from January 10, 2020, to February 28, 2020, in Hunan province. The patients were categorized into group 1 (≤ 19 days) and group 2 (> 19 days) based on the time from symptom onset to discharge. Cough during admission occurred more commonly in group 2 (68.4%) than in group 1 (53.1%, *p* = 0.042). White blood cell (*p* = 0.045), neutrophil counts (*p* = 0.023), Alanine aminotransferase (*p* = 0.029), Aspartate aminotransferase (*p* = 0.027) and Lactate dehydrogenase (*p* = 0.021) that were above normal were more common in group 2. Patients with single lesions were observed more in group 1(17.7%, *p* = 0.018) and multiple lesions observed more in group 2(86.8%, *p* = 0.012). The number of lobes involved (*p* = 0.008) in the CT score (*p* = 0.001) for each patient was all differences between the two groups with a statistically significant difference. Mixed ground-glass opacity (GGO) and consolidation appearances were observed in most patients. GGO components > consolidation appearance was more common in group 1 (25.0%) than in group 2 (8.0%) with a significant difference (0.015), GGO < consolidation was more common in group 2(71.1%, *p* = 0.012). From the logistic regression analysis, the CT score (OR, 1.223; 95% CI, 1.004 to 1.491, *p* = 0.046) and the appearance of GGO > consolidation (OR, 0.150; 95% CI, 0.034 to 0.660, *p* = 0.012) were independently associated with the hospitalization days. Thus, special attention should be paid to the role of radiological features in monitoring the disease prognosis.

## Introduction

Since several cases of pneumonia from an unidentified pathogen were reported in Wuhan, Hubei province, China in December 2019, a new type of coronavirus infectious pneumonia rapidly broke out, which was subsequently designated as a global health emergency by the World Health Organization (WHO)^1^. This novel coronavirus, identified using throat swab samples by the Chinese Center for Disease Control and Prevention, was named COVID-19 by the WHO.

According to the recent literature, the majority of patients presented as a lower respiratory tract inflammation, including fever, cough, myalgia, or fatigue^2–4^. The manifestations are similar to those of other viral pneumonia, such as Severe Acute Respiratory Syndrome (SARS) and Middle East Respiratory Syndrome (MERS)^5,6^. CT imaging has played an important role in the screening, primary diagnosis, and monitoring of patients with COVID-19, as lung imaging can provide evidence earlier than clinical manifestations. The imaging findings reported have been similar to those of typical viral pneumonia, but with certain significant features^7^. Several summary articles have focused on the clinical and radiological findings as well as the clinical outcomes, in which the outcomes were designated as severe and non-severe^8^. However, no research has yet focused on the prognosis in discharged patients. The purpose of this study was to evaluate whether a correlation exists between the early clinical, laboratory, and radiological findings and the different prognoses in discharged patients with COVID-19.

## Materials and methods

This study was approved by the Medical Ethical Committee of the second Xiangyang Hospital of Central South University (approval number: 2020004). The requirement for informed consent was waived, given the retrospective nature of this study, as per The Council for International Organizations of Medical Sciences guidelines of the second Xiangyang Hospital of Central South University.

### Patients

**Figure 1:**
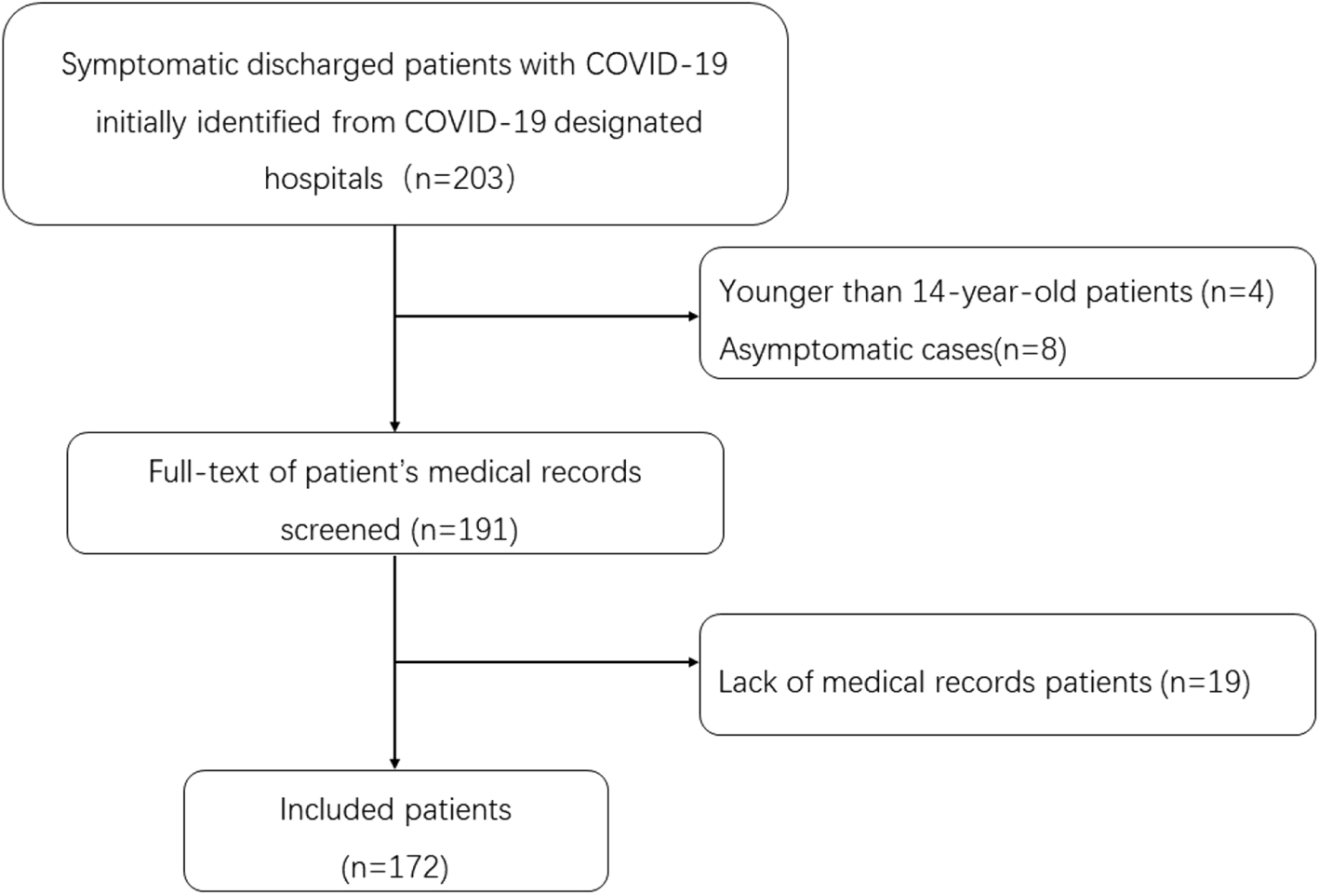
Flowchart of patient inclusion and exclusion in this study.

We retrospectively identified discharged patients with laboratory-confirmed COVID-19 from the electronic medical system and picture archiving and communication system (PACS) at two centers in Hunan following the Medical Ethical Committee of the second Xiangyang Hospital of Central South University approval (number: 2020004). A total of 172 patients (97 male and 75 females, with a mean age of 43.92 ± 14.85) discharged from January 10, 2020, to February 28, 2020, were included in this study with the following criteria: 1) patients had laboratory-confirmed COVID-19; 2) patients were discharged without readmission, and 3) patients underwent chest CT scanning and laboratory examination upon initial hospital admission. Patients who lack medical records and those younger than 14 were also excluded. A flowchart of the patient inclusion and exclusion in this study is presented in Fig. 1.

The diagnosis of COVID-19 based on the guideline (Trial version 5) from the China National Health Commission^9^ was as follows: (1) suspected patients with the detection of COVID-19 by real-time reverse-transcription–polymerase chain reaction; and (2) suspected patients with a virus gene sequence from the respiratory tract of a blood sample that strongly matched with COVID-19. The discharge criteria based on the guideline (Trial version 5) included: (1) the temperature returned to normal for more than three days and respiratory symptoms were significantly relieved; (2) the chest radiological images demonstrated significant improvement, and (3) a negative nucleic acid test was obtained for two consecutive respiratory pathogens of COVID-19 (sampling interval ≥ 1 day).

Based on the time from symptom onset to discharge, we designated two patient groups in our study: group 1 (onset-discharge days ≤ 19 days) and group 2 (onset-discharge days > 19 days).

### Data collection

The data of the demographic and clinical characteristics as well as laboratory findings were retrieved from electronic medical records by two trained physicians. The demographic information included sex, age, exposure history, smoking history, and underlying comorbidities. The clinical characteristics included the onset temperature and symptoms, the highest temperature and symptoms during the hospital stay, and the diagnosis upon admission.

### Image interpretation

All Digital Imaging and Communications in Medicine images from the cases were analyzed blindly and independently by three radiologists, with 5, 15, and 20 years of experience in interpreting chest CT images. The axial CT images and other multiplanar reconstructions in both the lung window (width: 1500 HU; level: − 700 HU) and mediastinal window (width: 350 HU; level: 40 HU) were reviewed. For each patient, features including the number of lesions (single or multiple); the number of lobes involved (ranging from 0 to 5); lesion density (pure ground-glass opacity [GGO], pure consolidation, and mixed GGO and consolidation); lesion morphology (presence of nodules, patchy shadowing, and linear shadowing); lesion distribution (bilateral or unilateral); and presence of bronchial abnormalities (including the air bronchogram sign and bronchodilation) were analyzed. A semi-quantitative CT score system was used to assess the involvement degree in each lung lobe: no involvement corresponded to a score of 0, 1% to 25% involvement corresponded to a score of 1, 25% to 50% involvement corresponded to a score of 2, 50% to 75% involvement corresponded to a score of 3, and more than 75% involvement corresponded to a score of 4^10^. Patients with mixed GGO and consolidation lesions were defined as two scenarios: GGO/consolidation > 1 referred to patients with GGO predominantly and GGO/consolidation < 1 referred to those with consolidation predominantly, as evaluated by the three radiologists. Following separate evaluations from each radiologist, the disagreements were discussed to reach a consensus.

### Statistical analysis

The continuous variables were expressed as the means and standard deviations (SDs), whereas the categorical variables were expressed as the counts and percentages in each category. We grouped patients into onset-discharge days ≤ 19 days and onset-discharge days > 19 days. Wilcoxon rank-sum tests were conducted using the continuous variables, whereas chi-square tests and Fisher’s exact tests were used for the categorical variables as appropriate. A *p*-value of < 0.05 was considered statistically significant. All of the variables with statistically significant differences between the two groups were analyzed using logistics regression to determine the independent influential factors. All statistical analyses were performed using SPSS (IBM).

### Ethical approval

All procedures performed in studies involving human participants were following the ethical standards of the institutional research committee and with the 1964 Helsinki declaration and its later amendments or comparable ethical standards.

### Informed consent

The requirement for informed patients’ consent was waived for this retrospective study referring to the CIOMS guideline.

## Results

### Demographic and clinical characteristics

**Figure 2:**
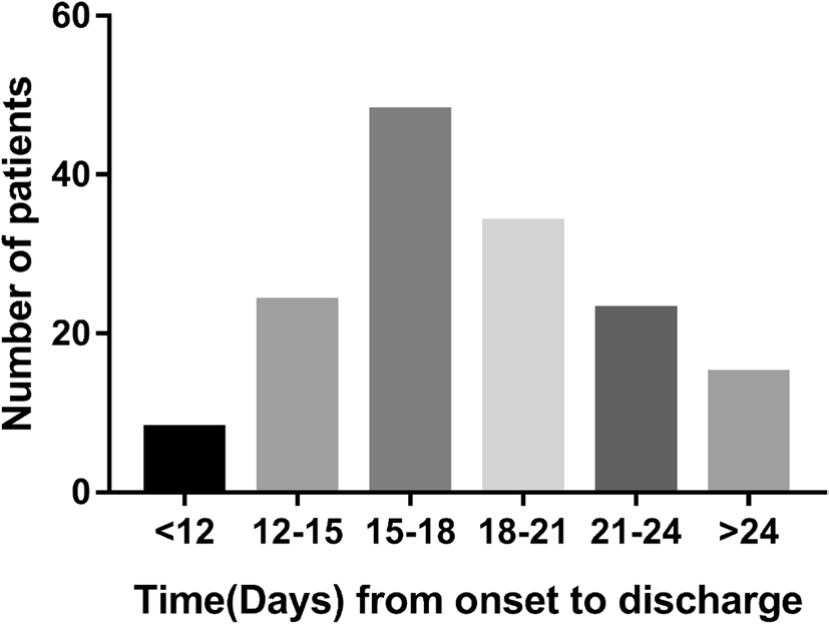
Hospitalization day and other time distributions for COVID-19 infected patients.

**Figure 3:**
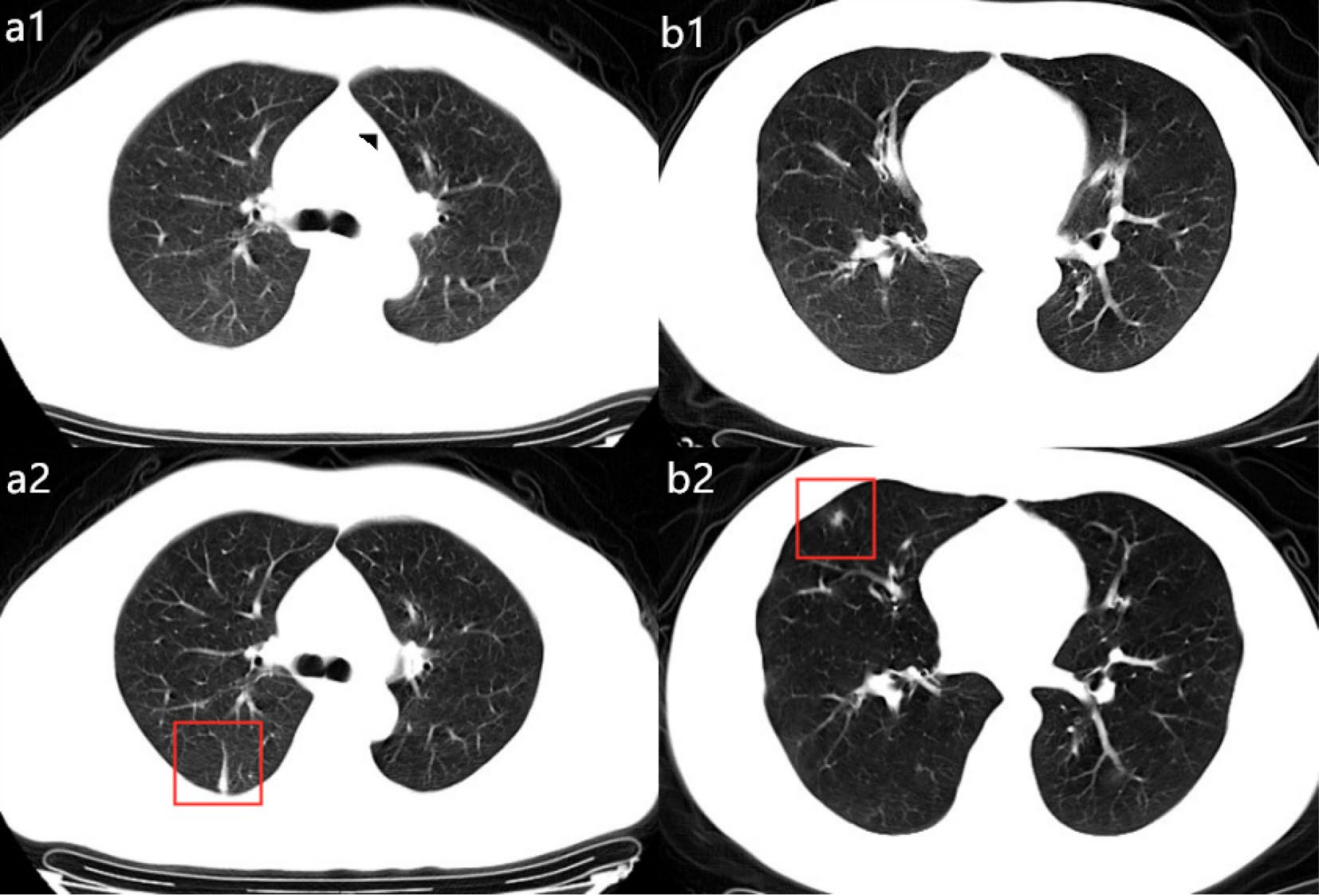
Examples of patients with admitting diagnosis as non-pneumonia in Group 2. (**a1**–**a2**) a 45-year-old man with confirmed with non-pneumonia COVID-19 and had hospitalization days of 16. Patients had fever at onset of disease, but normal CT images at the beginning of the admission (**a1**), at 4th day on admission, the follow-up CT images demonstrated inflammatory appearance as linear shadowing (**a2**). (**b1**–**b2**) a 31-year-old man diagnosed as non-pneumonia with COVID-19 who had cough at onset but normal CT images (**b1**). Total hospitalization time was 18 days. After 4 days admission, an irregular consolidate nodule can be seen in the right middle lobe from the follow-up CT images (**b2**).

Among the 172 discharged patients with confirmed COVID-19, the average time from symptom onset to discharge was 20.48 ± 8.22 (ranging from 3 to 50), with a median number of 19 days. The time from onset to the discharge of all patients is illustrated in Fig. 2. We divided the 172 patients into two groups based on the time from onset to discharge. A total of 96 patients (55.8%) who had time from onset-discharge no more than 19 days were assigned to group 1. A total of 76 patients (44.2%) who had longer time onset-discharge (more than 19 days) were assigned to group 2. The demographic and clinical characteristics of the patients according to the groups are summarized in Table 1. In the full cohort, there were 97 males (56.4%) and 75 females (43.6%), with a mean age of 43.9 years (SD: 14.8). There was no statistically significant difference in the age (*p* = 0.060), gender (*p* = 0.790) or current smoking history (*p* = 0.241) between the groups. History of direct exposure to Wuhan was documented in 48.8% of patients; the remaining 51.2% of patients had contact with people from Wuhan and there was no significant difference between the two groups (*p* = 0.376). There were 11 patients (11.5%) in group 1 and 6 patients (7.9%) in group 2 who had an admitting diagnosis of non-pneumonia (*p* = 0.437). Several non-pneumonia patients had longer hospitalization days owing to persistent symptoms or pneumonia occurrence during admission. Their CT images were initially normal but abnormalities occurred during admission (Fig. 3).

**Table 1:** Demographic and clinical characteristics of patients in this study.

	All patients (n = 172)	Group 1 (onset-discharge days ≤ 19) (n = 96)	Group 2 (onset-discharge days > 19) (n = 76)	*p* value
Age	43.92 ± 14.85	42.03 ± 14.52	46.32 ± 15.01	0.060
**Gender**				0.790
Male	97(56.4%)	55(57.3%)	42(55.3%)	
Female	75(43.6%)	41(42.7%)	34(44.7%)	
**History of exposure to Wuhan**^**1**^				0.376
Direct	84(48.8%)	44(45.8%)	40(52.6%)	
Indirect	88(51.2%)	52(54.2%)	36(47.4%)	
Current smokers	19(11.0%)	13(13.5%)	6(7.9%)	0.241
**Comorbidities**				
Hypertension	28(16.3%)	15(15.6%)	13(17.1%)	0.794
Diabetes	14(8.1%)	10(10.4%)	4(5.3%)	0.270
Coronary heart disease	3(1.7%)	1(1.0%)	2(2.6%)	0.584
Cerebrovascular diseases	7(4.1%)	3(3.1%)	4(5.3%)	0.701
Cancers^2^	1(0.6%)	0(0%)	1(1.3%)	0.442
More than one comorbidity	59(34.3%)	28(29.2%)	31(40.8%)	0.111
**Onset symptoms**				
Fever	106(61.6%)	61(63.5%)	45(59.2%)	0.562
Cough	81(47.1%)	39(40.6%)	42(55.3%)	0.056
Shortness of breath	6(3.5%)	3(3.1%)	3(3.9%)	0.770
More than one symptom	83(48.3%)	40(41.7%)	43(56.6%)	0.052
**Diagnosis on admission**				0.437
Simple infection	17(9.9%)	11(11.5%)	6(7.9%)	
Mild pneumonia	155(90.1%)	85(88.5%)	70(92.1%)	
**Symptoms on admission**				
Fever	114(66.3%)	63(65.6%)	51(67.1%)	0.838
Cough	103(59.9%)	51(53.1%)	52(68.4%)	0.042
Shortness of breath	18(10.5%)	8(8.3%)	10(13.2%)	0.305
More than one symptom	83(48.3%)	43(44.8%)	40(52.6%)	0.307
Highest temperature on admission	37.86 ± 0.8	37.84 ± 0.71	38.07 ± 0.85	0.097
Days from symptom onset to admission	6.02 ± 4.38	4.55 ± 3.46	7.88 ± 4.71	*P* < 0.001

Data are presented as medians ± SD and n (%).

^1^Direct history of exposure to Wuhan refers to those patients who entered into or stayed at Wuhan.

^2^Cancers referred to any malignancy. All cases were stable disease. *P* values denoted the comparison between Group 1 and Group 2.

The most common symptoms at the onset and during admission were fever (106, 61.6%; 114, 61.3%) and cough (81, 47.1%; 103, 59.9%). Cough during admission was more commonly observed in group 2(68.4%, *p* = 0.042).

### Laboratory and radiological findings

Table 2 presents the laboratory and radiological findings according to the groups at initial admission. White blood cell (*p* = 0.045) and neutrophil (*p* = 0.023) counts that were above normal were more common in group 2 (7, 9.2%; 8, 10.5%) than in group 1 (2, 2.1%; 2, 2.1%). Alanine aminotransferase (*p* = 0.029), Aspartate aminotransferase (*p* = 0.027) and Lactate dehydrogenase (*p* = 0.021) that above normal were more common in group 2. Except for 11 patients (11.5%) in group 1 and 6 patients (7.9%) in group 2 without abnormal findings in the chest CT at initial admission, the patients exhibited abnormal CT imaging features (Table 2). The majority of patients had multiple lesions (70.8%, 86.8%, *p* = 0.012) with bilateral distribution (65.6%, 78.9%, *p* = 0.055) in both groups 1 and 2. The mean number of lobes involved was 2.49 ± 1.71 in group 1 and 3.27 ± 1.75 in group 2 with a significant difference (*p* = 0.008). The mean score of the lung involvement exhibited a significant difference between group 1 (3.61 ± 3.00) and group 2 (5.80 ± 4.34, *p* = 0.001). The CT images of the majority of the patients represented mixed density (including GCO and consolidation) in both groups, but the proportion of GGO and consolidation differed in each patient.

**Table 2:** Laboratory and radiological findings of patients in this study.

	Days (≤ 19) from admission to discharge(n = 96)	Days (> 19) from admission to discharge(n = 76)	*p* value
**Laboratory findings**			
White blood cell count, > 10 * 10^9^/L	2(2.1%)	7(9.2%)	0.045
White blood cell count, < 4 * 10^9^/L	31(32.3%)	25(32.9%)	0.933
Neutrophil count, > 7 * 10^9^/L	2(2.1%)	8(10.5%)	0.023
Lymphocyte count, < 0.8 * 10^9^/L	19(19.8%)	13(17.1%)	0.653
Total bilirubin, > 21 mmol/L	6(6.3%)	11(14.5%)	0.073
Albumin, < 35 g/L	18(18.8%)	16(21.1%)	0.706
Alanine aminotransferase, > 40U/L	8(8.3%)	15(19.7%)	0.029
Aspartate aminotransferase, > 40U/L	7(7.3%)	14(18.4%)	0.027
Lactate dehydrogenase, > 225U/L	14(14.6%)	22(28.9%)	0.021
Procalcitonin, > 0.05 ng/mL	19(19.8%)	14(18.4%)	0.821
Blood urea nitrogen, > 8.2 mmol/L	3(3.1%)	3(3.9%)	1.000
Creatinine, > 104umol/L	2(2.1%)	2(2.6%)	1.000
D-dimer, > 1 mg/L	9(9.4%)	5(6.6%)	0.505
Radiological findings			
**Number of lesions, n**			
Single	17(17.7%)	4(5.3%)	0.018
Multiple	68(70.8%)	66(86.8%)	0.012
Average number of lobes involved, n	2.49 ± 1.71	3.27 ± 1.75	0.008
CT score, n	3.61 ± 3.00	5.80 ± 4.34	0.001
**Density, n**			
Pure ground-glass opacity	3(3.1%)	4(5.3%)	0.701
Pure consolidation	8(8.3%)	4(5.3%)	0.553
GGO/consolidation > 1	24(25.0%)	8(10.5%)	0.015
GGO/consolidation < 1	50(52.1%)	54(71.1%)	0.012
**Morphology, n**			
Patchy shadowing	28(29.2%)	28(36.8%)	0.286
Lineal shadowing	59(61.5%)	50(65.8%)	0.558
**Distribution, n**			
Unilateral	22(22.9%)	10(13.2%)	0.102
Bilateral	63(65.6%)	60(78.9%)	0.055
No abnormal findings, n	11(11.5%)	6(7.9%)	0.437

Data are presented as n(%).

*P* values denoted the comparison between group1 and group2.

**Figure 4:**
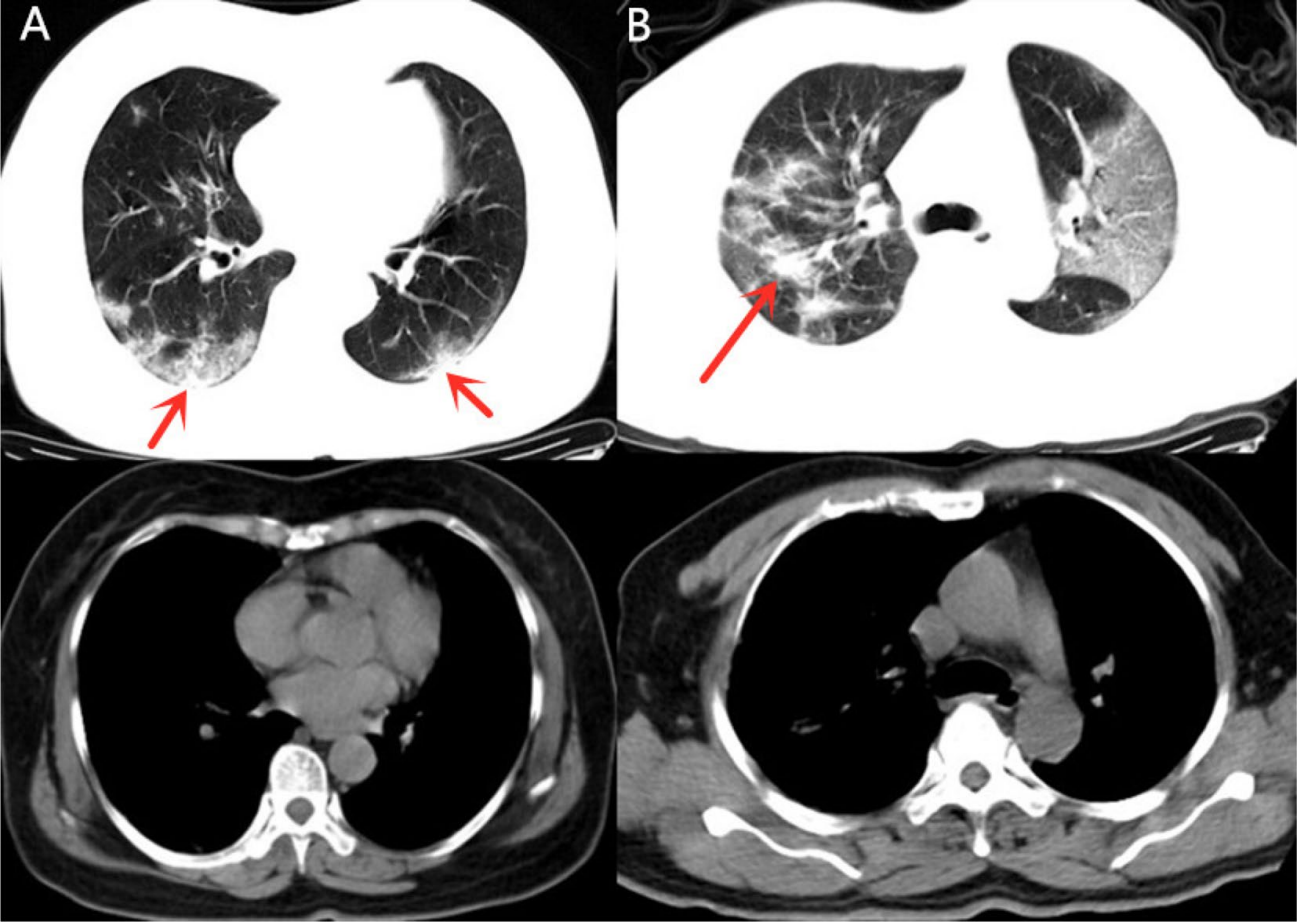
Example of patients with CT demonstrated as GGO > consolidation in Group 1. (**A**) 65-year-old female with confirmed COVID-19 pneumonia in Group 1 who had hospitalization time of 12 days. CT images demonstrated multiple mixed GGO and consolidation lesions with bilateral distribution, the consolidation component (arrow) was apparently less than GGO. (**B**) 53-year-old man in Group 1 with 12 admission days. Multiple mixed GGO and consolidation lesions with bilateral distribution were seen in his CT images, at the same time. The range of GGO components was apparently more than consolidation (arrow).

**Figure 5:**
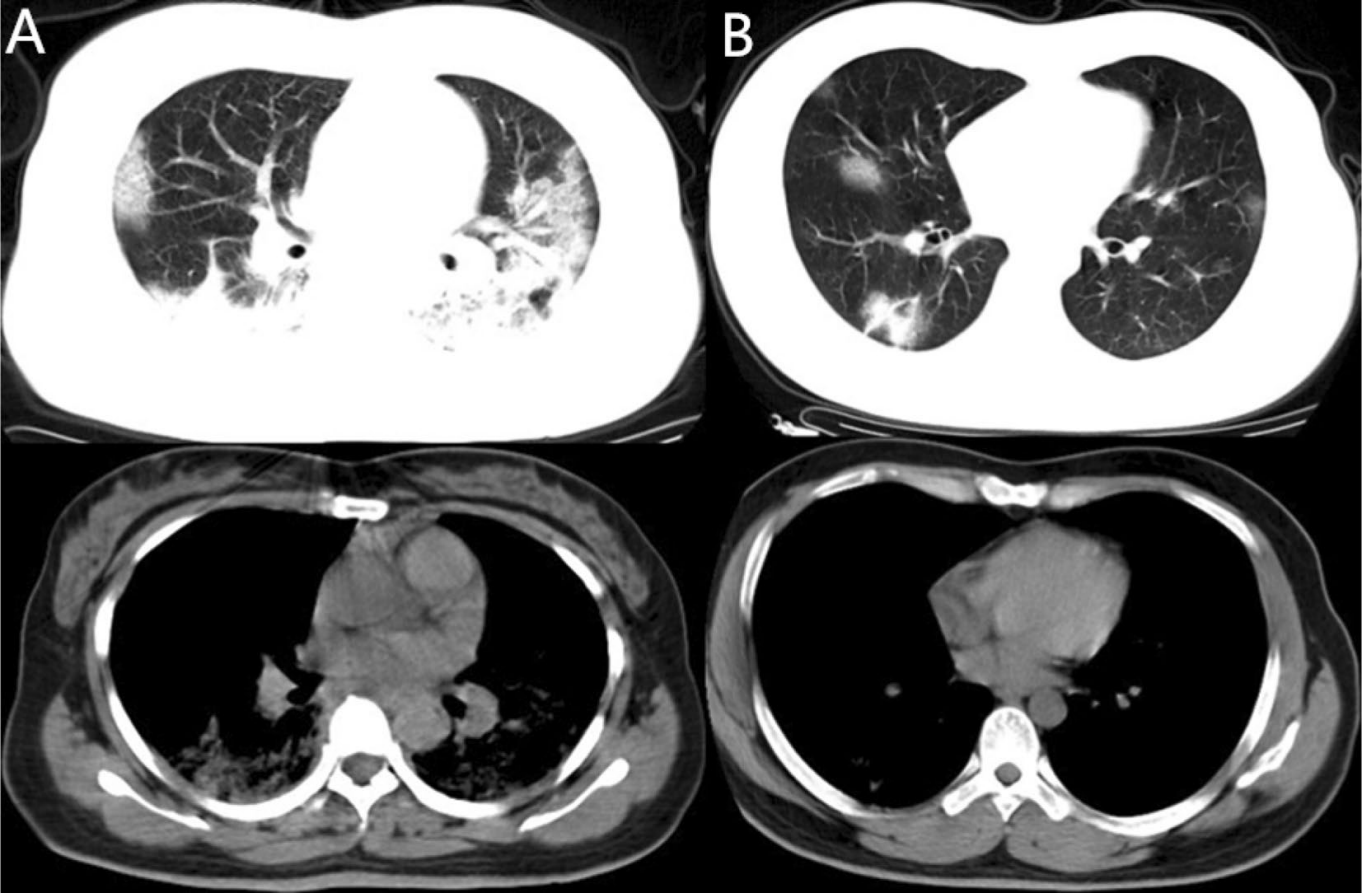
Example of patients with CT demonstrated as consolidation > GGO component in Group 2. (**A**) 29-year-old female confirmed with COVID-19 pneumonia in Group 2 who had hospitalization days of 19 days. CT images demonstrated multiple lesions with mixed density which were predominantly solid components (arrows). (**B**) 28-year-old male in Group 2 with hospitalization days of 19 days. CT images also demonstrated multiple mixed density lesions with predominantly solid components (arrows).

The demonstration of GGO components more than consolidation (Fig. 4) was higher in group 1 (24, 25.0%) than in group 2 (8, 10.5%), with a significant difference between the groups (*p* = 0.015). The demonstration of consolidation components more than GGO (Fig. 5) was higher in group 2 (54, 71.1%) than in group 1 (50, 52.1%) with significant difference (*p* = 0.012). The morphological features, including the presence of patchy shadowing (*p* = 0.286), and linear shadowing (*p* = 0.558) exhibited no significant difference between groups 1 and group 2.

### Clinical characteristics, laboratory findings, radiological findings, and prognosis

The variables from the clinical characteristics, laboratory findings, and radiological findings that were significantly different between the two groups were analyzed using logistic regression. The results are summarized in Table 3. CT score (OR, 1.223; 95% CI, 1.004 to 1.491, *p* = 0.046) of patients and appearance of GGO > consolidation (OR, 0.150; 95% CI, 0.034 to 0.660, *p* = 0.012) were associated with additional hospitalization days.

**Table 3:** Logistic regression analysis of risk factors and patients’ hospitalization days.

Factors	OR	95%CI	*P* value
Cough onset	1.190	0.5750–2.575	0.659
Alanine aminotransferase > 40U/L	1.118	0.287–4.348	0.872
Aspartate aminotransferase > 40U/L	2.886	0.589–14.146	0.191
Lactate dehydrogenase > 225U/L	0.499	0.151–1.652	0.255
Number of lobes involved	0.975	0.656–1.448	0.900
CT score	1.223	1.004–1.491	0.046
Lesions with GGO/consolidation > 1	0.150	0.034–0.660	0.012
Lesions with GGO/consolidation < 1	0.852	0.326–2.231	0.745

## Discussion

COVID-19 as an emerging infectious disease has aroused more and more attention since December 2019. Although the mortality rate of COVID­19 is considerably lower than that of SARS in 2003 and MERS in 2012, COVID-19 is highly infectious and could be a persistent health threat. Recently, an epidemiologic study from 44,672 laboratory-confirmed COVID-19 patients showed that most cases (81%) were classified as a mild type which included non-pneumonia and mild pneumonia^11^. Therefore, summarizing the characteristics of mild cases would be important and helpful to improve the understanding of COVID-19.

We retrospectively observed a group of 172 discharged patients with laboratory-confirmed COVID-19 to determine the correlation between clinical features and pneumonia prognosis. All patients had complete medical records including clinical information, laboratory and radiological data required for this study, and explicit inclusion and exclusion criteria for each patient were used to confirm the representative population. In our study, the average onset-discharge days of patients were 20.48 ± 8.22 (range, 3–50) days, and the median was 19 days. Besides, very little was found in the previous literature on the question about how to divide into groups based on the onset-discharge days in viral pneumonia. Thus, we used 19 days as a cut-off value to divide the discharged patients into two groups.

Cough (40.5%) was one of the most common symptoms during admission in patients with COVID­19 mild pneumonia. A meta-analysis found that patients with obvious respiratory symptoms were more likely to develop into poor prognosis^12^. In our study, the number of patients who had a cough during admission symptoms also showed a significant difference between the two groups. Furthermore, laboratory parameters of neutrophilia and raised LDH in COVID-19 patients have been shown by several studies^8,13,14^ to have statistical significance towards requiring ICU care. Similarly, Fan et al. found that neutrophilia, and raised LDH were predictive for ICU stay^15^. It would be obvious to clinicians that patients requiring ICU stay would have a much longer hospitalization than those not requiring ICU. Our results described coincident results in patients with a longer hospitalization. Although neutrophilia and raised LDH could not directly predict a longer hospitalization, these differences may be relevant to it.

A further study with more focus on the mechanism of inflammatory factors in COVID-19 patients is therefore suggested. In terms of the cause of neutrophilia, the finding will doubtless be much scrutinized, but there are some dependable conclusions, such as the use of steroids (IV hydrocortisone, prednisolone), excessive inflammation, and immune suppression caused by COVID-19 infection and resultant cytokine storm. On the one hand, neutrophils are pro-inflammatory cells with a range of antimicrobial activities, which can be potentiated by virus-related inflammatory factors, such as raised interleukin-6 and interleukin-8.

Secondly, COVID-19-associated liver injury is defined as any liver damage during the duration of COVID-19 in patients. In our study, we showed that liver injury was more frequent in Group 2, such as elevated ALT and AST. These results demonstrated the relationship between impaired liver function and longer hospitalization days. Qin et al. found that a high level of AST or ALT might be the prediction of a worse prognosis in COVID-19 patients^16^. Also, Lei et al. analyzed COVID-19 patients for liver enzymes and found that elevated AST was strongly associated with the mortality risk^17^. However, the underlying mechanism of abnormal liver enzymes and the relationship between COVID-19 and liver injury is still unclear. Further study would be beneficial to reveal the possible reasons. Surprisingly, the number of non-pneumonia patients had no significant difference in the two groups with different onset-discharge days. We double-checked their medical record. There was no obvious mistake in data collection. It is difficult to explain this result, but it might be related to the selection of cut-off values. Furthermore, a part of patients without pneumonia had obvious and persistent symptoms in our cohort. The hospitalization days of them were more than that of patients with a short duration of symptoms.

In terms of radiological findings, the current study found that the number of involved lung lobes and CT scores of patients with longer hospitalization days were significantly higher than that of patients with shorter hospitalization days. Non-specific features such as the number of lesions, morphology, distribution, and bronchus involved in our cohort. These findings are similar to those of previous radiological studies of patients with COVID-19 pneumonia^18,19^. However, compared with the early characteristics of lesions that had the majority of pure ground-glass opacity (GGO) or consolidation^20^, the characteristics of patients in our cohort showed the majority of mixed lesions. To investigate the possible reasons for the discrepancies, we calculated the proportion of GGO with consolidation or consolidation with GGO in mixed lesions, respectively. These results showed that the greater proportion of GGO and the greater proportion of consolidation in mixed lesions had obvious differences between the two group patients. Further investigation using binary logistic regression also revealed that a greater proportion of GGO was associated with the hospitalization days. It is difficult to clearly explain these results, but these might be related to several aspects. On the one hand, the GGO is dissipated more easily than the consolidation so that patients would help patients recover quickly from the COVID-19 infection. There are similarities between the attitudes expressed by the dissipation of consolidation in this study and those described by the previous studies. Han et al. showed that most patients with consolidation lesions dissipated into GGO, then GGO continuously into aborsption^21^.

On the other hand, the severity type of patients was included in our cohort. CT finding of the greater proportion of consolidation, high CT scores were a feature of them. Although these findings cannot be found in all patients, we reasonably reckoned that the great proportion of consolidation would be harmful for the lung function of patients, even be related to the severity of the disease, so that patients would have longer hospitalization days.

There were some limitations in our study. Firstly, as a relatively small and three-center study, it is so difficult to avoid selection bias, and the characteristics of enrolled patients may not be representative. Secondly, due to the average age of the patients in our cohort was around 40 years old, whether these results are generalizable to COVID-19 mild pneumonia patients in children or aged greater than 70 years old needs further evaluation. Secondly, as lung biopsy specimens were unable to analyze in this study, the relationship between histopathological and radiological findings remains to be investigated. Finally, the diagnosis and discharge criteria in this study were based on the guideline (Trial version 5) from the China National Health Commission, which is the latest version at the moment when study was done. With the COVID-19 pandemic, clinical care for COVID-19 patients has been changed in China or in other country. Until January 2021, China National Health Commission proposed the latest guideline (version 8)^22^ for COVID-19 maintaining the discharge criteria unchanged. However, from the clinical management proposed by World Health Organization^23^, which recommended that symptomatic patients can be discharged at the 10 days after symptom onset, plus at least 3 days without symptoms (fever and respiratory symptoms).

## Conclusions

In conclusion, this was a retrospective, multicenter, and comprehensive study focused on the impact factors in hospitalized COVID-19 mild pneumonia outcomes. Chest CT could help prompt diagnosis and monitor disease progression, GGO/consolidation > 1 in mixed lesions was associated with shorter hospitalization days. Special attention should be paid to the role of radiological features in monitoring disease prognosis.
